# Study on Wear and Fatigue Performance of Two Types of High-Speed Railway Wheel Materials at Different Ambient Temperatures

**DOI:** 10.3390/ma13051152

**Published:** 2020-03-05

**Authors:** Lei MA, Wenjian WANG, Jun GUO, Qiyue LIU

**Affiliations:** 1 School of Mechanical Engineering, Xihua University, Chengdu 610039, China; 2 Tribology Research Institute, State Key Laboratory of Traction Power, Southwest Jiao Tong University, Chengdu 610031, China

**Keywords:** alpine region, high-speed wheel and rail materials, temperature, wear, fatigue damage

## Abstract

The wear and fatigue behaviors of two newly developed types of high-speed railway wheel materials (named D1 and D2) were studied using the WR-1 wheel/rail rolling–sliding wear simulation device at high temperature (50 °C), room temperature (20 °C), and low temperature (−30 °C). The results showed that wear loss, surface hardening, and fatigue damage of the wheel and rail materials at high temperature (50 °C) and low temperature (−30 °C) were greater than at room temperature, showing the highest values at low temperature. With high Si and V content refining the pearlite lamellar spacing, D2 presented better resistance to wear and fatigue than D1. Generally, D2 wheel material appears more suitable for high-speed railway wheels.

## 1. Introduction

Railways play a vital role in the development of rail transportation. The environmental climate has a certain impact on wheel/rail systems exposed to the open air. Especially in China, wheel and rail materials may serve under extreme temperature conditions due to torridity and severe cold. For example, the highest and lowest temperature experienced by the Qinghai–Tibet railway during operation can reach +40 °C and −45 °C, respectively [1,2]. With the activation of the Haerbin–Dalian railway, the world’s first high-speed railway running through an alpine region, safe railway operation in the alpine region has attracted more and more attention [3]. 

**Figure 1:**
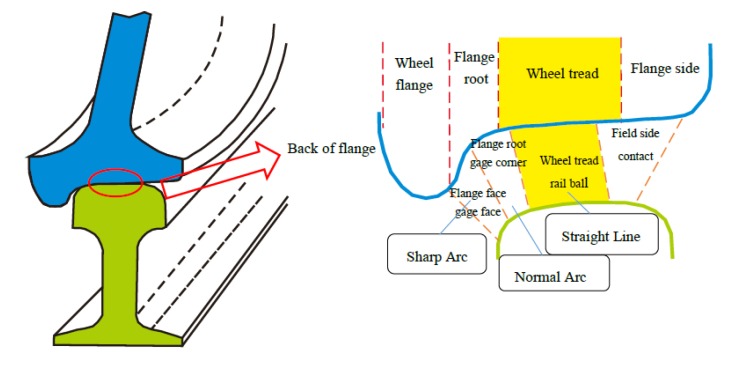
Contact points of the rail and wheel in a variety of paths [4].

Wear and fatigue are common damages of wheel and rail materials during railway operation. Wear (especially at the wheel tread and rail ball interface under straight-line running conditions, as studied in this paper and shown in Figure 1) will directly lead to the reduction of rail height, thus causing plastic deformation of materials and failure of surface materials [4]. Common wear mechanisms include abrasive wear, adhesive wear, delamination wear [5]. Fatigue derives from the formation of cracks and the detachment of material from the surface due to the repetitive application of alternating forces [6,7], which by first causing defects or surface cracks, may eventually destroy some components [8]. Especially in high-speed railways operating in extremely hot and cold climates, the mechanical properties of temperature-sensitive wheel and rail materials will change at certain extreme temperatures. Furthermore, friction performance and safety can be affected during train operation [9]. In recent years, brittle fractures in steel structures caused by fatigue have occurred from time to time. One of the characteristics of brittle fracture compared with ordinary strength failure is that brittle fracture often occurs in cold winter [10]. In China, 60–80% of rail fracture accidents occur from November to March [11], and in Japan, 83 of the 121 rail failures recorded by a study occurred from November to February [12]. Shank et al. investigated many accidents and found that the change of steel’s notch sensitivity was the main cause of accidents that occurred at extreme temperatures [13]. It was also found that with the gradual decrease of temperature, fractures in railway material will change from the ductile shear fracture mode to the brittle cleavage fracture mode, causing the decline of fracture toughness, impact energy, and plasticity [14,15,16,17,18].

As early as the 1980s, some scholars have studied the material properties of ferritic steel at low temperature by means of mechanical experiments and material preparation methods [19,20,21,22,23,24]. The results showed that extreme temperatures can cause the degradation of various ferritic steel properties, such as strength and toughness, before the formation of macroscopic cracks, affecting the safety of wheel/rail services [25,26,27]. Also, Zhu, Lyu et al. [28,29] conducted a study on wheel/rail tribology in a low-temperature environment by using a pin-and-disk testing machine. The experimental results showed that the low-temperature environment had a great impact on the friction performance of the wheel/rail materials. Nevertheless, research on the performance of wheel and rail materials in a low-temperature environment is limited. Therefore, it is very necessary to select wheel and rail materials suitable for cold climates and analyze their wear and fatigue performance under extreme temperature conditions.

## 2. Materials and Methods 

**Figure 2:**
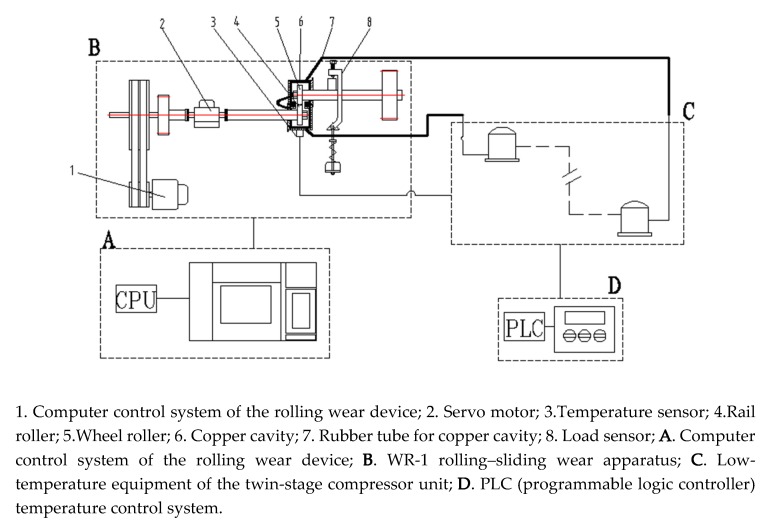
Scheme of the WR-1 low-temperature simulation system.

The WR-1 rolling–sliding wear apparatus with a temperature-changing device was used to evaluate the wear and rolling contact fatigue (RCF) properties of D1 and D2 wheel rollers with friction pair of U71Mn rail material under different temperature conditions. As shown in Figure 2, the apparatus is composed of two rollers which served as a wheel roller (upper specimen) and as a rail roller (lower specimen). The rail roller (4) is driven by a DC motor, while the wheel roller (5) is driven via a pair of gears. A normal force (from 0 to 2000 N) is applied by a compressed spring to the upper specimen. Load sensors (8) are used to measure the tangential friction force and normal force at the wheel/rail interface (measurement error: ±5%). A low-temperature environment is created by double compressors (C) which make the refrigerant (Freon) to recirculate in the copper tube. A rubber tube (7) and copper cavity (6) connect the WR-1 rolling–sliding wear apparatus (B) to the low-temperature environment, resulting in a low temperature in the copper cavity (6) due to the flow of refrigerant. The wheel and rail rollers are installed in the copper cavity (6). High ambient temperature is reached using an electric heating tape entwined around the copper cavity (6). A digital temperature sensor (3) is fixed into the copper cavity (6) and the temperature is monitored in the cavity in real time. A PLC (Programmable Logic Controller) temperature control system (D) is used to stabilize the temperature in the copper cavity at a set value. The temperature error in the copper cavity is ±2 °C. During the experiment, the ambient temperature of the wheel and rail rollers are kept stable.

**Figure 3:**
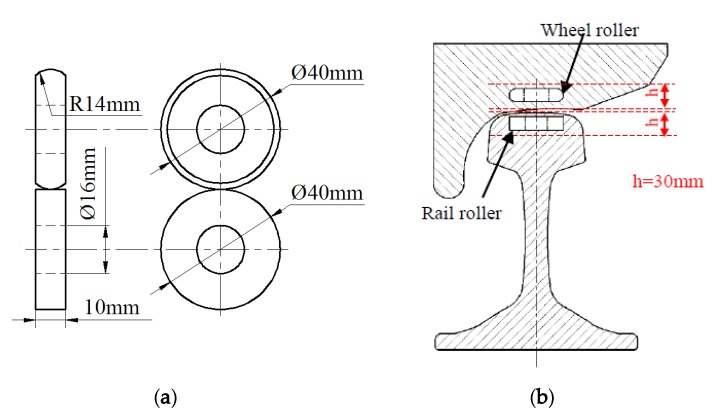
Dimensions and sampling position of wheel and rail rollers. (**a**) Dimensions; (**b**) sampling position.

The dimensions of wheel roller (upper specimen) and rail roller (lower specimen are shown in Figure 3a. The diameter of wheel and rail rollers was 40 mm. The wheel and rail rollers were cut from the wheel tread and railhead (0–30 mm from the surface), respectively, as shown in Figure 3b. The samples were molded into 40 mm-diameter rollers through machining processes such as turning and drilling, and all rollers were polished to surface roughness (Ra) of approximately 0.15 μm. Before testing, each roller was thoroughly cleaned by ultrasonic cleaning for 15 min. The chemical compositions of wheel and rail materials in weight percentage are given in Table 1.

In the test, the rotational speed of the rail roller was 400 r/min, and the number of cycles was 3.84 × 10^5^. The normal force between the wheel and the rail rollers was about 150 N. The slip ratio was 0.91%. In addition, the experiments were performed at high temperature (50 °C), room temperature (RT: 20 °C), and low temperature (−30 °C).

**Figure 4:**
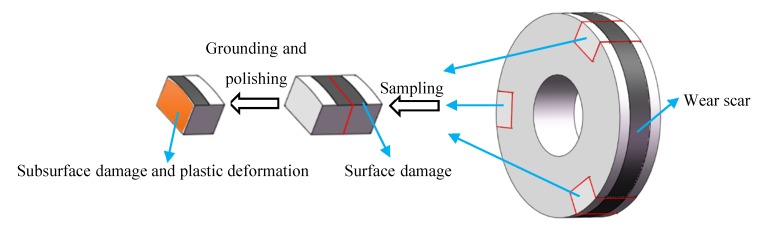
Sampling positions of surface and cross sections of the wheel/rail rollers for examination after wear testing.

**Figure 5:**
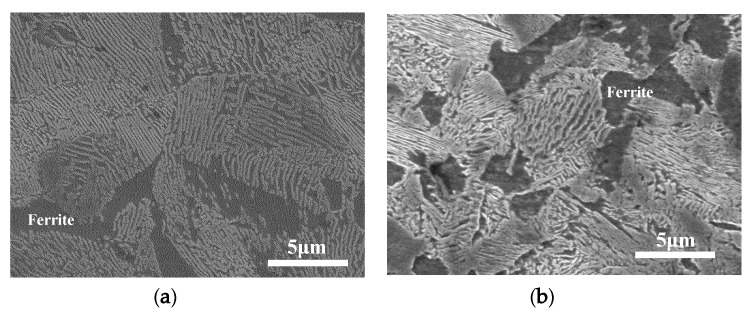
Metallographic microscope images of wheel materials. (**a**) D1; (**b**) D2.

The rollers were ultrasonically cleaned in ethanol, dried, and weighed using an electronic balance (JA4103, measurement accuracy: 0.0001 g) before and after testing. Wear loss of the wheel and rail rollers was determined by calculating the mass loss. Figure 4 shows the sampling positions of the surface and cross sections in the wheel/rail rollers. Three pieces of 1 mm length separated by 120° were taken from each roller. Each section was cut along the rolling direction by wire-cutting processing, mounted in resin, ground with 2000-grit abrasive paper, polished with 0.5 μm diamond, and etched with 4% Nital. The plastic deformation of wheel and rail rollers was characterized using optical microscopy (OM) (OLYMPUS BX60M, Tokyo, Japan). Metallographic microscopy and surface and subsurface damages were inspected using scanning electronic microscopy (SEM) (QUANTA200 FEI, Eindhoven, Netherlands). Metallographic microscopy images of D1 and D2 materials are shown in Figure 5. It can be seen that pearlite composed of cementite and ferrite presents a lamellar structure, and the Pearlite lamellar spacing in D1 is larger than that in D2.

## 3. Results

### 3.1. Wear Loss

**Figure 6:**
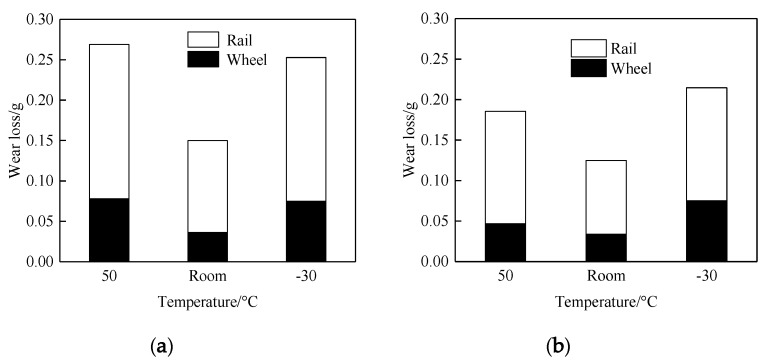
Wear loss of wheel/rail rollers. (**a**) D1; (**b**) D2.

Figure 6 shows the wear loss of wheel and rail rollers under different temperatures. It is clear that the wear loss of the D2 friction pair was slightly lower than that of the D1 friction pair, and both the wear losses of the D1 and D2 friction pairs were higher at 50 °C and −30 °C than at room temperature. The wear loss of D1 at 50 °C was not different from that at −30 °C, while that of D2 at −30 °C was higher than that at 50 °C. The results showed that D2 had better wear resistance than D1, especially at 50 °C. For both wheel materials, the wear resistance at RT was the best, followed, in order, by wear resistance 50 °C and at −30 °C. At the same time, the wear loss of the rail roller was obviously larger than that of the wheel roller, which is consistent with plastic deformation of the material occurring in the testing process.

### 3.2. Hardness and Plastic Deformation

**Figure 7:**
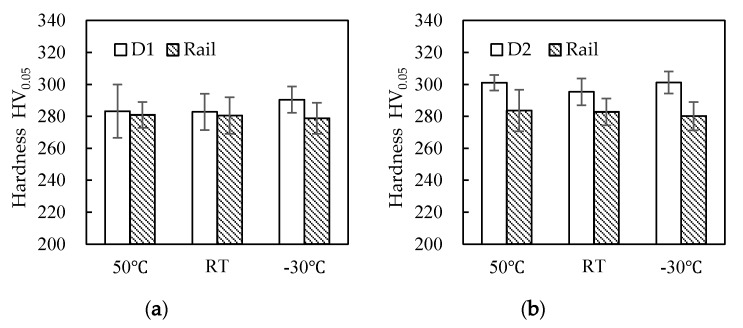
Surface hardness of wheel and rail samples before the experiment. (**a**) D1 friction pair; (**b**) D2 friction pair.

**Figure 8:**
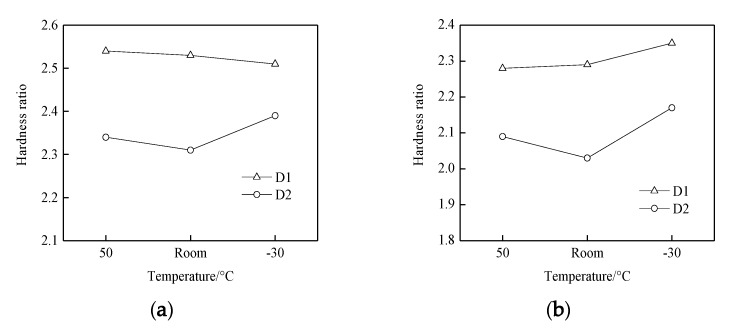
Surface hardness ratios of wheel and rail samples of values measured after and before the experiment:.(**a**) ratios of hardness values of the wheel measured after the experiment and before the experiment; (**b**) ratios of hardness values of the rail measured after the experiment and before the experiment.

Hardness tests were conducted on the wheel and rail samples before and after the experiment. It was found that the surface hardness of D1 samples was about 285 HV_0.05_ before the experiment, that of D2 samples was 300 HV_0.05_, and that of the rail samples was 280 HV_0.05_ (Figure 7). When comparing the surface hardness of the wheel and rail samples before and after the experiment (Figure 8), the hardness of the wheel and rail materials after the experiment was more than twice that before the experiment. The wheel and rail samples of the D1 friction pair showed a significantly greater surface hardness ratio than those of the D2 friction pair, indicating that surface hardening of the D1 friction pair was greater than that of the D2 friction pair. At the same time, the wheel surface hardening was greater than that of the rail, which caused a high wear of the rail samples. With the decrease of temperature, the surface hardening of D1 decreased, while that of the rail increased. For the D2 friction pair, the surface hardening of the wheel and rail samples was greater at 50 °C and −30 °C than at room temperature; it significantly increased at −30 °C. The results showed that the resistance to hardening of D2 was stronger than that of D1; however, D2 wheel material was more significantly affected by temperature, and surface hardening was the lowest at room temperature.

**Figure 9:**
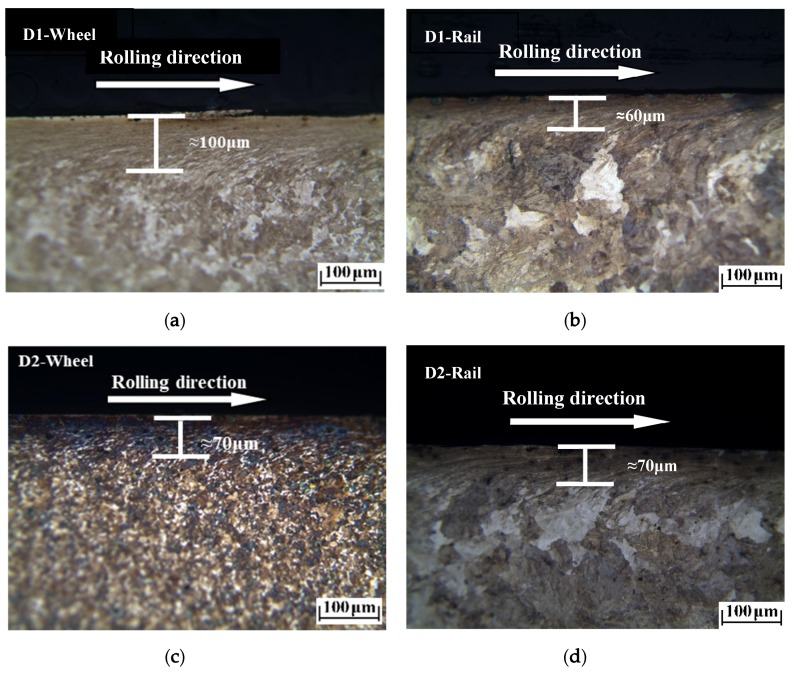
Plastic deformation of wheel and rail rollers at 50 °C: (**a**) D1 wheel; (**b**) rail of D1 friction pair; (**c**) D2 wheel; (**d**) rail of D2 friction pair.

**Figure 10:**
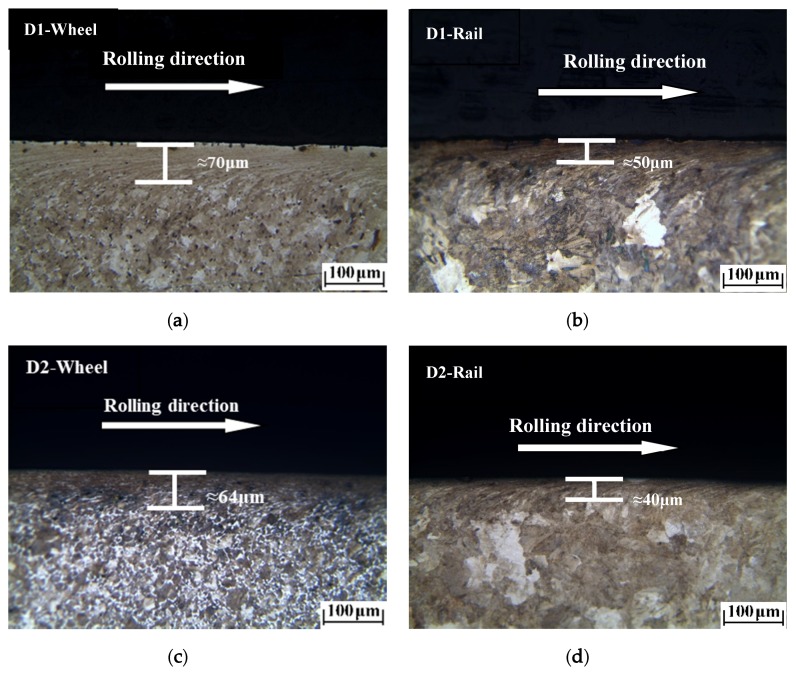
Plastic deformation of wheel and rail rollers at room temperature: (**a**) D1 wheel; (**b**) rail of D1 friction pair; (**c**) D2 wheel; (**d**) rail of D2 friction pair.

**Figure 11:**
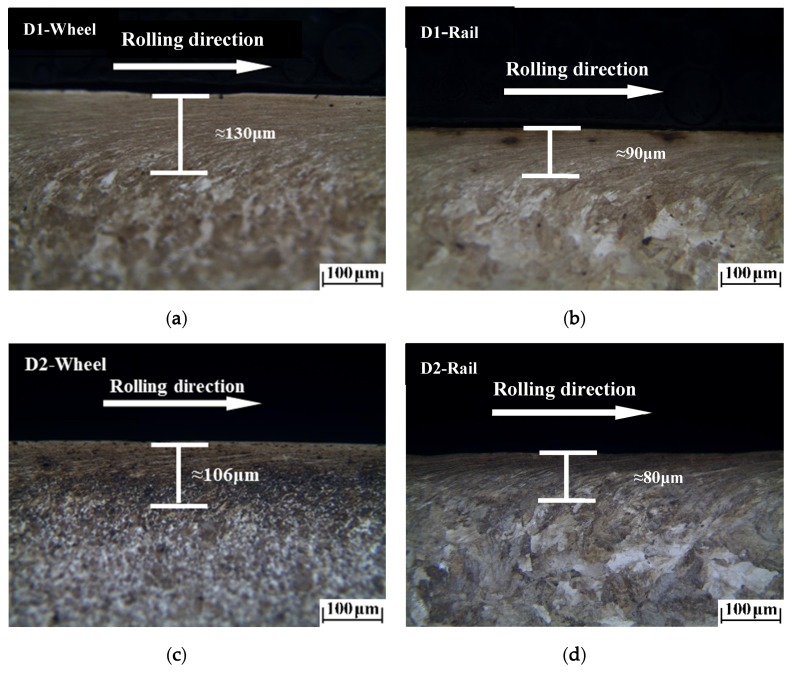
Plastic deformation of wheel and rail rollers at −30 °C: (**a**) D1 wheel; (**b**) rail of D1 friction pair; (**c**) D2 wheel; (**d**) rail of D2 friction pair.

The plastic deformation morphologies were obtained by metallographic treatment of the wheel and rail samples. In Figure 9, Figure 10 and Figure 11, it can be seen that at different temperatures, the D1 and D2 friction pairs showed the same change trend in plastic deformation thickness, with the greatest plastic deformation at −30 °C, and the least plastic deformation at room temperature. At the same temperature, the plastic deformation thickness of D1 was larger than that of D2, which means better deformation resistance of D2 and is consistent with the results of surface hardening. Compared to the wheel roller, the rail roller showed strong resistance to deformation, and the greatest plastic deformation occurred at −30 °C, similar to what observed for the wheel roller.

### 3.3. Surface Damage

**Figure 12:**
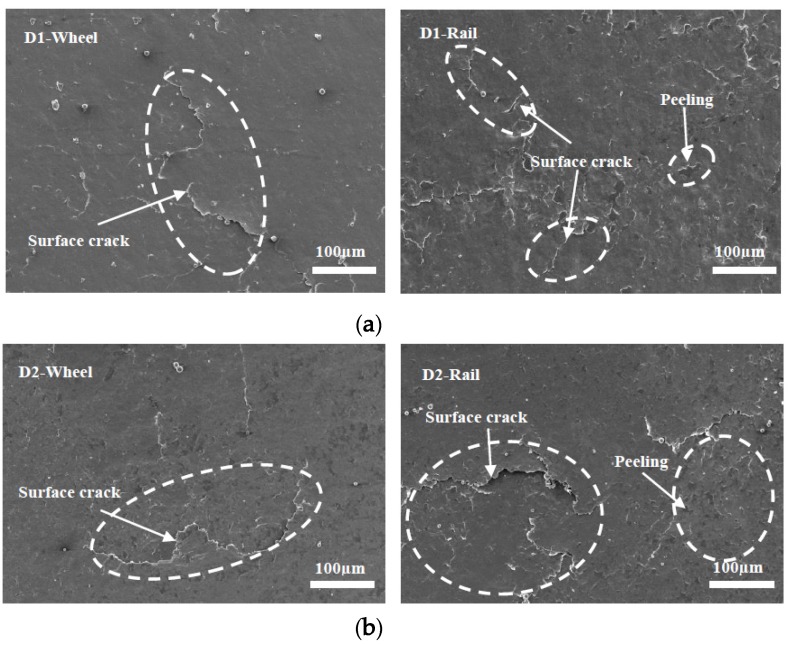
Micrographs of the worn surfaces of the wheel and rail rollers at 50 °C. (**a**) D1; (**b**) D2.

**Figure 13:**
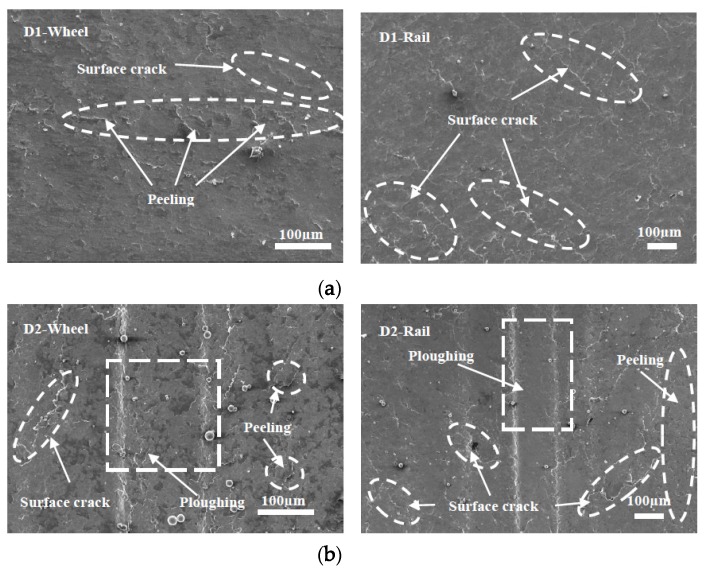
Micrographs of the worn surfaces of the wheel and rail rollers at room temperature. (**a**) D1; (**b**) D2.

**Figure 14:**
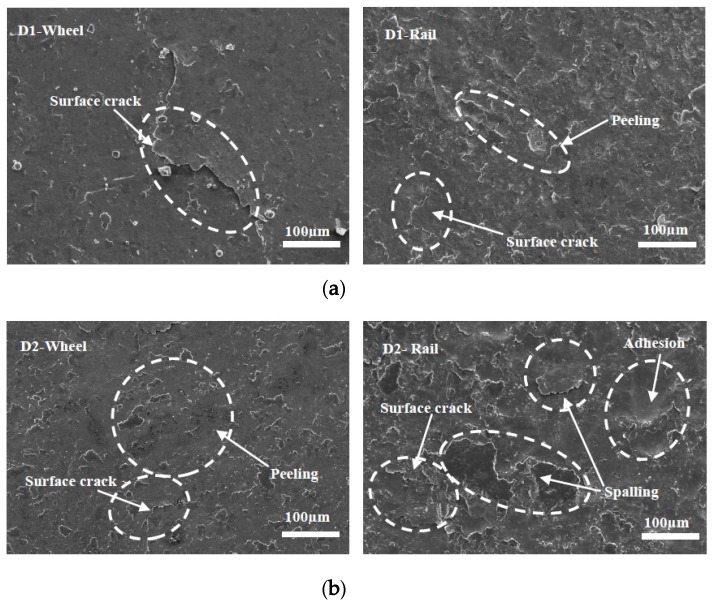
Micrographs of the worn surfaces of the wheel and rail rollers at −30 °C, (**a**) D1; (**b**) D2.

Micrographs of the worn surfaces of the wheel and rail rollers at different temperatures are shown in Figure 12, Figure 13 and Figure 14. It can be seen in Figure 12 that at 50 °C, peeling and surface crack damage occurred on the surfaces of the D1 friction pair, while for the D2 friction pair, damage was mostly due to large surface cracks. The surface damage was mild at room temperature, and peeling was the main damage on the surface of the D1 wheel roller, while surface cracks were predominant on the rail roller (Figure 13). Moreover, mixed damage consisting of ploughing, peeling, and surface cracks was present on the surfaces of the D2 friction pair at room temperature. When the temperature decreased to −30 °C, the surface damage aggravated (Figure 14). For the D1 friction pair, surface fatigue crack was the main type of damage on the surface of the wheel roller, while slight peeling and surface cracks were predominant on the rail surface. The surface damage of the D2 wheel roller was relatively mild and was mainly due to peeling, while a combination of large adhesion areas, spalling, and surface cracks was observed on the rail surface. With the temperature declining, the surface damage gradually increased and transformed from surface cracks and peeling to spalling and adhesion damage. Overall, the surface damage of the D2 wheel and rail samples was more serious than that of the D1 wheel and rail samples.

### 3.4. Subsurface Damage

**Figure 15:**
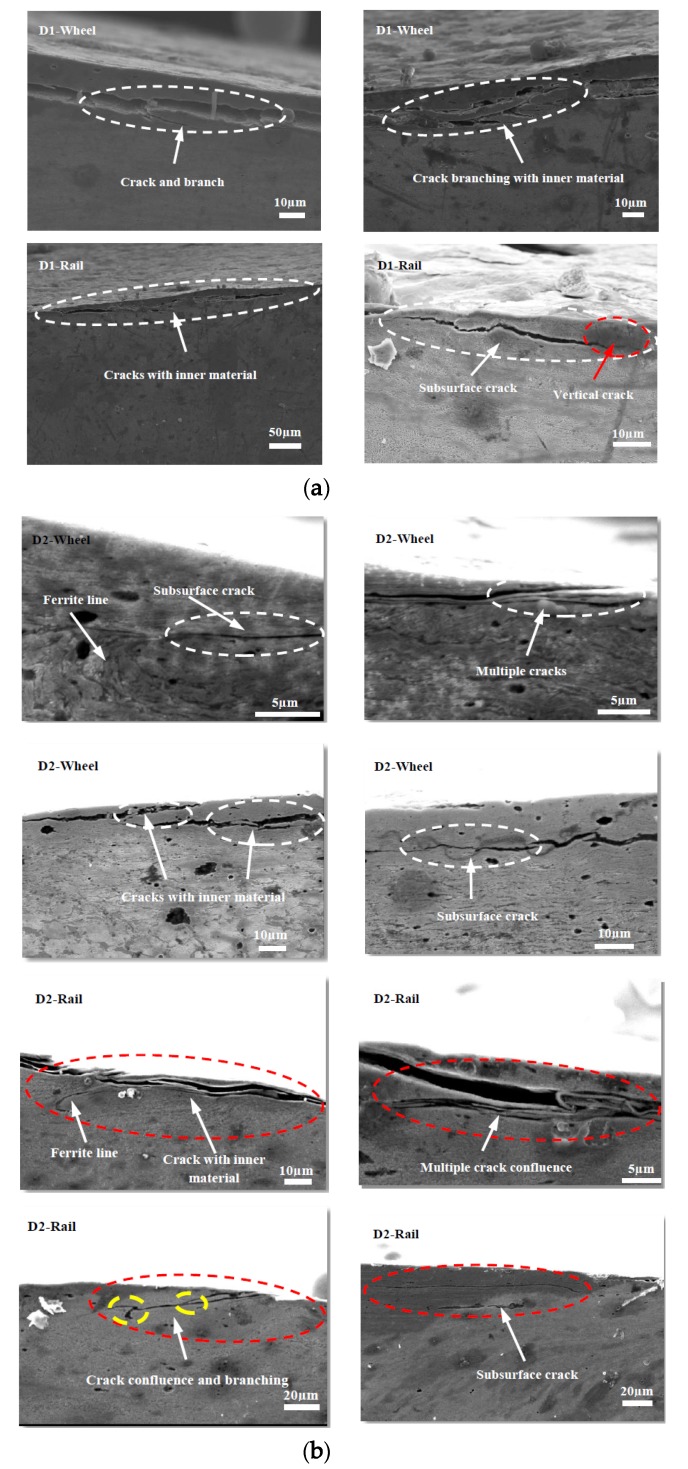
SEM micrographs showing the types of damage of wheel and rail rollers at 50 °C. (**a**) D1; (**b**) D2.

**Figure 16:**
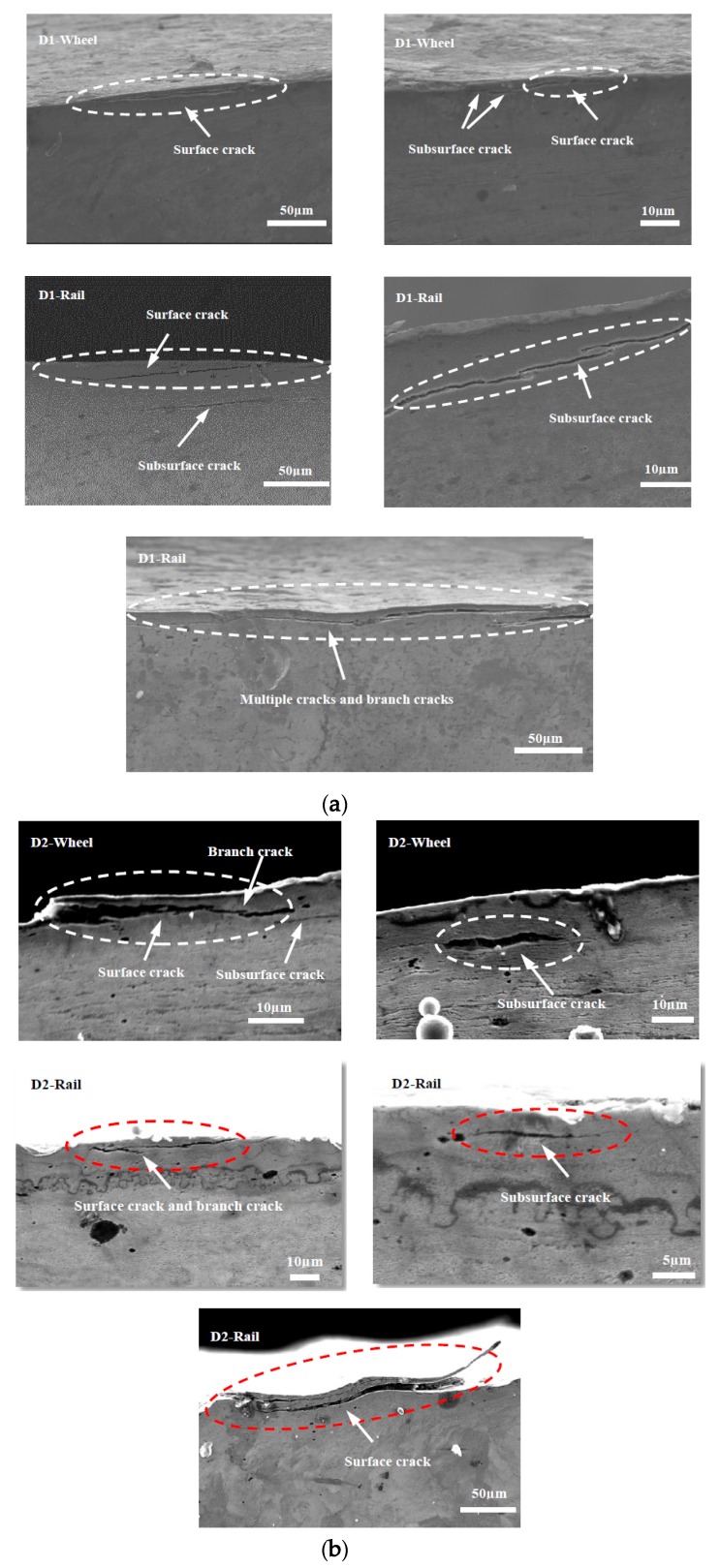
SEM micrographs showing the types of damage of wheel and rail rollers at room temperature. (**a**) D1; (**b**) D2.

**Figure 17:**
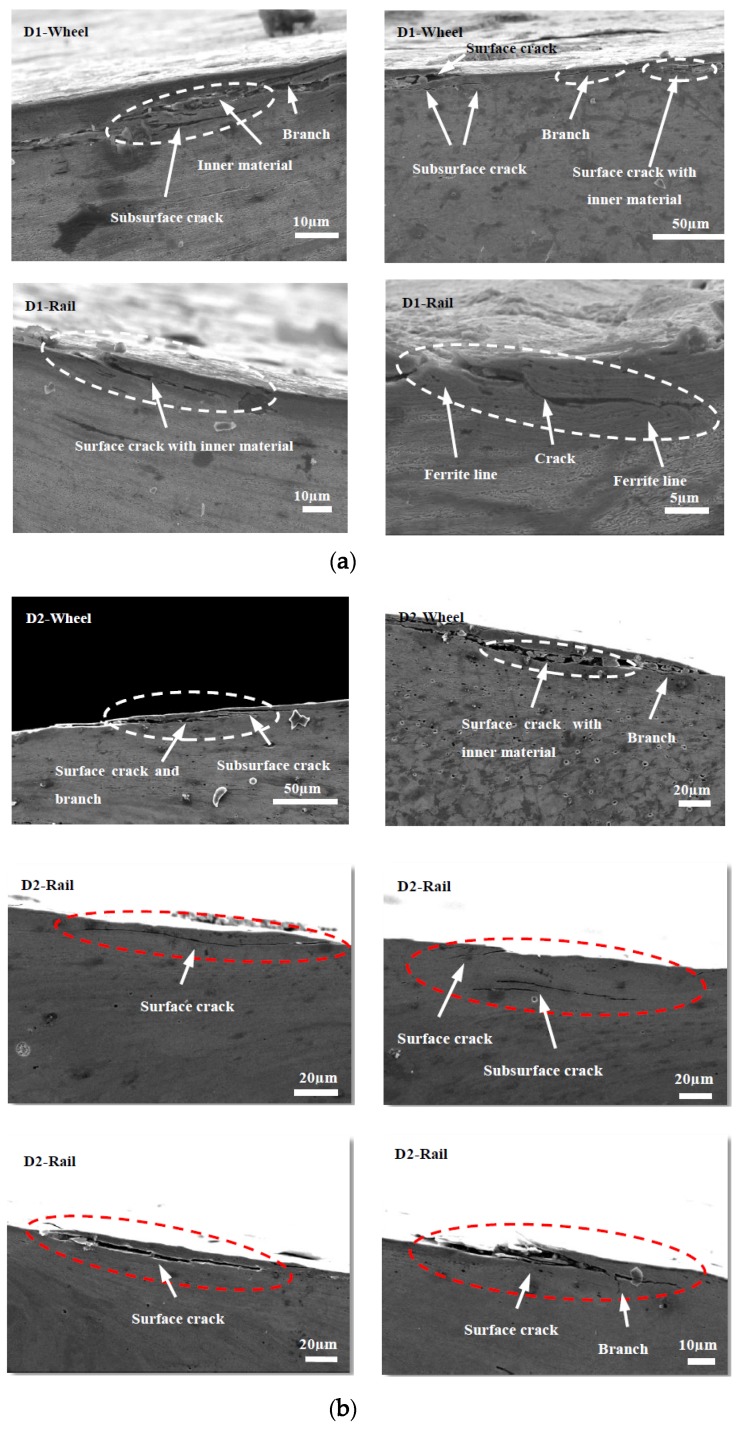
SEM micrographs showing the types of damage of wheel and rail rollers at −30 °C. (**a**) D1; (**b**) D2.

Figure 15, Figure 16 and Figure 17 show the subsurface damage of the D1 and D2 friction pairs at 50 °C, room temperature, and −30 °C, respectively. At 50 °C and −30 °C, the fatigue crack length and angle of the D1 and D2 wheel and rail rollers were larger compared to those at room temperature. It can be seen from Figure 15 and Figure 16 that the subsurface fatigue crack of the D2 wheel roller was relatively short and deep compared with that of the D1 wheel roller at 50 °C and room temperature. The long fatigue crack of D1 propagated along the surface, while the short fatigue crack of D2 extended slightly inward, with a small angle, while at −30 °C the subsurface damage of the D1 and D2 wheel rollers was accompanied by subsurface crack and a long internal crack in the material (Figure 17). For the rail roller, the main damage at room temperature was a long crack extending parallel to the surface and a subsurface crack. Under the conditions of 50 °C and −30 °C, there was a lamellar material inclusion inside the surface crack of the rail roller (Figure 15b); the crack extended to the inside and branched, so that subsurface crack damage increased compared with what observed at room temperature, becoming especially serious at −30 °C. The results showed that the subsurface damage of D1 was serious compared to that of D2 at the same temperature. Subsurface damage was severely affected by low temperature and less severely by 50 °C; the slightest subsurface damage to the two kinds of wheel materials was observed at room temperature.

## 4. Discussion

The ambient temperature has a remarkable influence on the wear and fatigue properties of D1 and D2 rollers. Our experimental results showed that wear loss, surface hardening, and plastic deformation of D1 and D2 wheel materials were particularly serious at low temperature. Meanwhile, D2 exhibited good wear resistance and hardening resistance compared to D1. Except for the ploughing damage on the surface of the D2 wheel roller at room temperature, surface damage type and degree of the two kinds of wheel materials were basically the same. D2 exhibited a slightly smaller surface damage and fatigue resistance than D1, and the surface and subsurface damage was severe at −30 °C.

Under the condition of 50 °C and −30 °C, both the fatigue crack angle and the fatigue crack length in the D1 and D2 friction pair increased, compared to those at room temperature. Thus the surface material was more easily damaged at 50 °C and −30 °C, and the fatigue damage was serious, indicating poor fatigue resistance performance. At the same temperature, the fatigue crack damage generated on the subsurface of the D2 material was slightly less and shorter in length than that on D1, which indicated that the anti-fatigue properties of D2 were slightly better than those of D1. In regard to the composition and alloying elements of the tested materials, the differences in Si and V contents led to different performances of the D1 and D2 wheel materials. In pearlitic steels used for wheel materials, the combination of V and C can form a stable VC structure, which improves the strength of ferrite the material yield strength, and the low-temperature toughness; however, V can also increase the ferrite phase volume fraction and reduce the tensile strength. Meanwhile, the addition of Si refines the pearlite lamellar spacing and reduces the ferrite volume fraction, which significantly increases the yield strength and tensile strength [30]. Generally, Si and V enhance a material’s hardness and tensile strength and refine pearlite lamellar spacing, which improves wear resistance and hinders crack propagation. Therefore, the D2 material, containing 2.6 times as much Si and 14 times as much V as D1, presented increased resistance to wear, hardening, and fatigue.

As for the fatigue characteristics of wheel and rail materials, they can be analyzed from a microscopic and kinematic perspective. With the action of Cyclic loading caused branch cracks, and inclusions inside cracks appeared during fatigue crack propagation in D1 and D2 wheel rollers. The subsurface damage of the rail material was more serious than that of the wheel material. Especially at 50 °C and −30 °C, crack growth, internal inclusions, and subsurface cracks in the rail material were more serious than at room temperature, indicating that the temperature had a significant impact on the damage of the wheel and rail materials. When the material was locally subjected to large contact stress during periodic cycle loading, surface cracks developed forming secondary branches or connections with subsurface cracks, thus extending inside the material. This resulted in further deformation and crushing of the cracks, causing accumulation of material inside the cracks. In some of these cracks, the inclusions accelerated crack propagation to the surface, material fracture under high cyclic load, and surface peeling and spalling. At the same time, the connection of several adjacent surface cracks or of surface and subsurface cracks led to crack growth, material surface peeling, or lamellar material accumulation inside the cracks, which progressed with cyclic loading (Figure 15a). The fatigue cracks generated in the D1 and D2 wheel materials also branched during cyclic loading (Figure 15, Figure 16b and Figure 17). 

## 5. Conclusion

(1) Ambient temperature has an obvious influence on the wear and fatigue properties of D1, D2, and rail materials. At low and high temperatures, wear loss, plastic deformation, and subsurface damage were more severe than at room temperature; they were the greatest at low temperature. while Surface damage aggravated as temperature decreased. In regard to the surface hardness ratio, the D1 friction pair showed slight decline with the temperature decreased, while the D2 friction pair presented the lowest surface hardening at low temperature, slightly better at high temperature, and the mildest at room temperature.

(2) With high content of Si and V which refined the pearlite lamellar spacing, the D2 wheel material showed high strength and hardness before testing, which led to a low wear loss and light subsurface damage. Despite the mild surface damage of the D1 wheel material, the resistance to wear and fatigue of the D2 wheel material was better than that of D1.

(3) Comparing the wear and fatigue properties of the two wheel materials, the D2 wheel material appears more suitable for high-speed railway wheels, and its performance is optimal at room temperature.

## Figures and Tables

**Table 1:** Chemical composition of the wheel and rail rollers (wt%).

Roller	C	Si	Mn	P	S	Cr	Al	V
D1 wheel	0.52	0.26	0.73	0.0060	<0.002	0.25	0.023	≤0.005
D2 wheel	0.54	0.68	0.70	0.0047	0.0013	0.055	0.011	0.07
Rail	0.65~0.75	0.1~0.5	0.8~1.3	≤0.025	≤0.025	-	-	-
